# Design of a medical record review study on the incidence and preventability of adverse events requiring a higher level of care in Belgian hospitals

**DOI:** 10.1186/1756-0500-5-468

**Published:** 2012-08-29

**Authors:** Annemie Vlayen, Kristel Marquet, Ward Schrooten, Arthur Vleugels, Johan Hellings, Elke De Troy, Frank Weekers, Neree Claes

**Affiliations:** 1 Hasselt University, Faculty of Medicine, Patient Safety Group, Agoralaan Building D, Room D58, Diepenbeek 3590, Belgium; 2 Ziekenhuis Oost-Limburg, Schiepse Bos 6, Genk, 3600, Belgium; 3 Centre for Health Services and Nursing Research, K.U.Leuven, Kapucijnenvoer 35/4, Leuven, 3000, Belgium; 4 ICURO, Handelstraat 82, Brussels, 1040, Belgium; 5 Jessa Ziekenhuis, Stadsomvaart 11, Hasselt, 3500, Belgium

**Keywords:** Medical errors, Adverse events, Medical records, Medical audit, Intensive care units, Hospitals

## Abstract

**Background:**

Adverse events are unintended patient injuries that arise from healthcare management resulting in disability, prolonged hospital stay or death. Adverse events that require intensive care admission imply a considerable financial burden to the healthcare system. The epidemiology of adverse events in Belgian hospitals has never been assessed systematically.

**Findings:**

A multistage retrospective review study of patients requiring a transfer to a higher level of care will be conducted in six hospitals in the province of Limburg. Patient records are reviewed starting from January 2012 by a clinical team consisting of a research nurse, a physician and a clinical pharmacist. Besides the incidence and the level of causation and preventability, also the type of adverse events and their consequences (patient harm, mortality and length of stay) will be assessed. Moreover, the adequacy of the patient records and quality/usefulness of the method of medical record review will be evaluated.

**Discussion:**

This paper describes the rationale for a retrospective review study of adverse events that necessitate a higher level of care. More specifically, we are particularly interested in increasing our understanding in the preventability and root causes of these events in order to implement improvement strategies. Attention is paid to the strengths and limitations of the study design.

## Findings

### Background

An important indicator of patient safety is the rate of adverse events in hospitals. An *Adverse event* can be defined as (1) an unintended injury or complication, (2) which results in disability at discharge, death or prolongation of hospital stay, and (3) is caused by healthcare management (including omissions) rather the patient’s disease [1-5]. Although all medical errors should be a concern, errors that either result in serious consequences for patients or that are preventable are of particular concern. A substantial number of adverse events is detected among unintended Intensive Care Unit (ICU) admissions and readmissions. Unplanned Intensive Care Admission (UIA) is an existing clinical indicator, used in several countries on a regular basis. It was developed and implemented in Australia, in a close collaboration between the Australian and New Zealand College of Anaesthetists (ANZCA) and the Australian Council on Healthcare Standards (ACHS) and recommended as a measure of patient safety (“avoidable incidents in anaesthesia”) and the effectiveness of care (“lack of planning”) [6].

To estimate the incidence and preventability of adverse events requiring ICU (re)admission, we conducted a systematic review including medical record review studies [7]. A total of 27 studies were included, of which 14 studies addressed unplanned ICU admissions due to anaesthetic or surgical adverse events, eight studies investigated adverse events on general wards and five studies focused on ICU readmissions. Due to study heterogeneity, meta-analysis of the data was not appropriate. Results showed that the percentage of surgical and medical adverse events requiring ICU admission ranged from 1.1% to 37.2%. ICU readmissions varied from 0% to 18.3%. Preventability of the adverse events varied from 17% to 76.5%. Consequences of the adverse events included a mean length of ICU stay that ranged from 1.5 days to 10.4 days for the patient’s first stay in ICU and mortality percentages between 0% and 58%. The large variation in study outcomes can be explained by methodological diversity. The included studies varied in sample size, applied different methods of screening and only three out of 27 studies used a multi-center design. On the other hand, clinical diversity was high because of population mix and variation (or absence) of definitions on adverse outcomes. As a conclusion, we suggest that planning of future studies should aim to standardize terminology and measures of outcomes (standard taxonomy) and to apply more explicit study designs in order to allow for comparisons across studies.

Several nationwide studies describe the use of medical record review to measure the occurrence of adverse events in hospitals [1-4,8-11]. ‘Unplanned transfer from general to intensive care’ is often used as a criterion (‘trigger’ or clue) to uncover adverse events and medical errors [2,4,8,10,12]. The positive predictive value (PPV) reflecting the reliability of this screening criterion varies from 1.9% [10] - 3.1% [4] to 6.5% [2]. Basically, the process of medical record review involves a multi-stage record review in which in the first stage the records are assessed by trained nurses for the presence of a predefined set of explicit criteria, indicating a potential adverse event. Each record that is positive for one or more criteria is forwarded to the next stage and reviewed by physicians for confirmation. The assessment of causation and preventability is performed using classification scales. Modifications in methodology among these studies involve different screening criteria, the reviewers’ education, definitions, timeframe of included events or the assessment of causation and preventability [1,2,4,9,11].

In Belgium, the occurrence of adverse events has never been assessed through medical record review. Retrospective analysis of the national hospital discharge dataset of all Belgian acute hospitals for the year 2000 estimated the incidence of adverse outcomes to be 7.12% for medical and 6.32% for surgical hospital stays, with a high variability between hospitals [13].

Currently, there are 194 Belgian hospitals, of which 105 acute, 66 psychiatric and 23 long-term care hospitals. Acute hospitals consist of university hospitals, general hospitals ‘with university character’ and other non-university hospitals. Belgium has seven university hospitals, one for each medical school that offers the entire medical education. The Flemish region of Belgium has 55 acute hospitals. The province of Limburg, which is a part of the Flemish region, has seven acute hospitals, of which two hospitals with a university character [14]. This multicenter study is initialized in the province of Limburg and aims at identifying preventable adverse events that contributed to the transfer of patients to a higher level of care using the method of chart review. This study is funded by ‘Limburg Sterk Merk’, a foundation of public use that supports healthcare and economic development projects.

It is not in the purpose of this study to detect all the adverse events in the inpatient records. An important goal is to make a clear distinction between the causality (errors) and the consequences (patient harm) of the adverse events. Rating preventability is important in understanding the system specific aspects of health care processes in order to design preventive or mitigating barriers.

The objectives of this multicenter study are to:

1. Determine the incidence of adverse events requiring a transfer to a higher level of care;

2. Assess the preventability of these adverse events;

3. Assess the clinical impact of these events;

4. Evaluate the adequacy and completeness of the patient charts;

5. Evaluate the use of medical record review as an auditing tool.

Spin-off studies will be undertaken to:

1. Explore the clinical and system specific causes of these adverse events and gain insight into potential preventive strategies (Root Cause Analysis); and

2. Assess the costs of the adverse events (separate cost study).

### Methods/design

#### Design and setting

A retrospective cohort study will be undertaken in six acute hospitals in the province of Limburg. All acute hospitals from the province of Limburg were invited to participate in this study. Six out of seven hospitals confirmed their participation and gave permission to access their patient charts.

#### Type of participants and record selection

To minimize selection bias, all records of the patients being transferred to a higher level of care and being discharged from or deceased in the hospital during the inclusion period (November 2011 - May 2012), irrespective the hospital admission date of the patient, will be screened for the occurrence of adverse events. In practice, record selection is based on (1) (re)admission to the Intensive Care Unit from other care units in the hospital providing lower intensity care, (2) an intervention by a Medical Emergency Team (MET) due to an unanticipated change in the patient’s clinical status or (3) a redo procedure within 24 hours for ICU patients. Considering that record selection is not based on routine hospital registration, hospitals were instructed to select the cases using a uniform selection form.

Because of their specific nature, patients admitted on neonatal or maternal ICUs will be excluded. Also planned admissions to the ICU from the operation room (major elective surgery) and ICU admissions directly from the emergency department will be excluded. As the included hospitals have no pediatric ICUs, only patients from the age of 16 or over will be included.

Starting from January 2012, patient records will be reviewed in a multistage review process by a research nurse (holder of a specialization degree in Intensive Care/ Emergency care), a physician (holder of a specialization degree in Anesthesiology/ Urgent and Emergency Medicine) and a clinical pharmacist. Chart review will be performed once the entire -closed and complete- record is available to the reviewers. A complete record consists of a medical (including laboratory and radiology results), nursing and pharmaceutical record. However, medical reports that are found to be incomplete or ambiguous are also included in the review process, as exactly in these cases the possibility of containing adverse events might be higher [15]. The review period is accomplished when all the included records are reviewed. It is expected that the period between record selection and review is relatively short and is largely dependent on (1) the length of stay from the time of transfer to a higher level of care and (2) the date of availability of the medical records. It is also expected that the structure of the records will not be uniform in all participating hospitals.

### Power calculation

The main (numerical) objective of this study is to estimate an overall incidence rate of adverse events (number of adverse events/patient days at risk). It is not in the aim to compare the results of the participating hospitals.

The precision of this estimate will be provided by a 95% confidence interval. The sample size of this study is determined in order to guarantee a sufficiently narrow confidence interval for the estimate.

**Figure 1:**
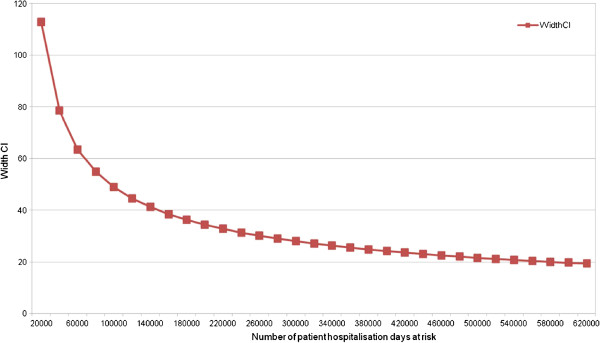
**Sample size calculation.** Abbreviations: *CI*, Confidence Interval; *AE*, Adverse Event.

From a pilot study of two months, 66 patients with one or more adverse events leading to a higher level of care were detected for 44 165 days at risk (149 per 100 000 patient days at risk) (Figure 1). At this rate, a sample size of 100 000 patient days at risk would provide a confidence interval of approximately 20% (+/− 10% around the estimate). As the total yearly number of in- patient days (excluding palliative, neonatal, pediatric and one day-stay admissions) for the six participating hospitals is 76 0057 (year 2010), this sample size corresponds to an inclusion period of six to seven months.

Different levels of clustering can be considered in this study: hospital level, ward level, pathology level, individual patient level. Since little is known about the impact of these different levels of clustering, clustering is not considered in calculating the sample size.

#### Outcome measures

Primary outcome measures are the number of patients transferred to a higher level of care because of an adverse event -or a combination of adverse events- per 100 000 patient days at risk, and the number of preventable adverse events in comparison with the number of adverse events. The number of patient days at risk is calculated as the total number of hospitalization days in the participating hospitals during the study period (excluding palliative, neonatal, pediatric and day-stay admissions).

Secondary outcomes are the type of event (operative, procedural, diagnostic, therapeutic, drug/ intravenous fluid or system issue), attributable causes and consequences of the events (level of patient harm, mortality and length of stay in hospital and ICU).

Independent variables are presented in a non-exhaustive list in Table 1.

**Table 1:** Independent variables

– Primary diagnosis for admission to the hospital
– Patient history
– Patient age (in years); year of birth
– Gender
– Number of prescribed drugs before hospital admission
– Admission day and time to ICU
– ICU admission source (location/ providers of care)
– Length of total hospital stay (prior to ICU admission) (LOS) (in days)
– Length of ICU stay (in days)
– Outcome in the ICU (discharge, mortality)
– Acute Physiology and Chronic Health Evaluation (APACHE) II
Patient complexity and mortality risk are defined according to the All Patient Refined Diagnosis Related Groups, which is calculated based on patient diagnosis, procedure, and age using a scale of 1 (least complex/lowest risk) to 4 (most complex/highest risk).
– Quality and completeness of the medical records
– Time measures screening process

#### Definitions

The definitions are adopted from previous adverse events studies [4,10,11,16-18]. They are described in Table 2. 

**Table 2:** Definitions

	
*Adverse event*	(1) An unintended injury or complication, which results in (2) disability at discharge, death or prolongation of hospital stay, and (3) is caused by healthcare management (including omissions) rather than the patient’s disease [4].	
*Unintended injury*	Refers to any disadvantage for the patient that leads to prolonged or strengthened treatment, temporary or permanent (physical or mental) impairment or death [11].	
*Disability*	Refers to temporary or permanent impairment of physical or mental function attributable to the adverse event (including prolonged or strengthened treatment, prolonged hospital stay, readmission, subsequent hospitalization, extra outpatient department consultations or death) [11].	
*Causation*	Refers to injury caused by health care management including acts of omission (inactions) i.e. failure to diagnose or treat, and acts of commission (affirmative actions) i.e. incorrect diagnosis or treatment, or poor performance [11].	
*Health Care Management*	Includes the actions of individual hospital staff as well as the broader systems and care processes and includes both acts of omission (failure to diagnose or treat) and acts of commission (incorrect diagnosis or treatment, or poor performance) [10].	
*Preventable Adverse Event*	An injury that is caused by medical intervention or management (rather than the disease process) and either prolonged hospital stay or caused disability at discharge, where there was enough information currently available to have avoided the event using currently accepted practices [16].	
*Higher Level of Care*	A higher level of care may include:	
	1.	An unplanned transfer to an Intensive Care Unit,
	2.	An intervention of a Medical Emergency Team or
	3.	A redo procedure within 24 hours of ICU patients.
*Intensive Care Units (ICUs)*	Hospital units providing continuous surveillance and care to actually ill patients (Mesh definition).	
	E.g. medical and surgical ICUs, for example Medium Care, Coronary Care Units, Pediatric ICUs and Respiratory Care Units.	
*Planned ICU admissions*	Admissions of patients expected to arrive on the ICU.	
	E.g. routinely scheduled post-surgery admissions or transfers directly to the ICU from outside hospitals.	
*Unplanned ICU admissions*	All patients unexpectedly admitted to the intensive care unit from a lower level of care in the hospital during the study period. If a patient experienced more than one unplanned ICU admission during his/her hospital stay, each unplanned admission is included in the analysis (adapted from Baker, 2009) [17].	
*Patient harm*	Unintended physical injury resulting from or contributed to by medical care that requires additional monitoring, treatment or hospitalization, or that results in death (IHI) [18].	

#### Data collection and review process

**Figure 2:**
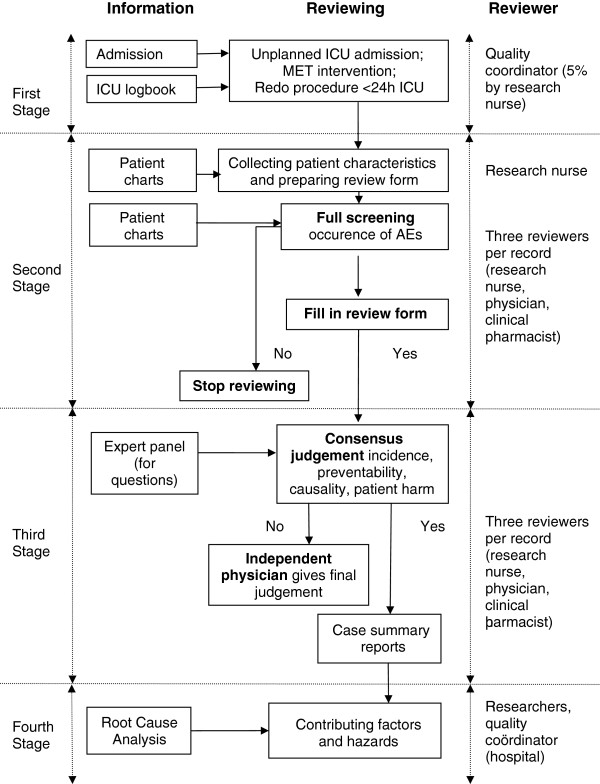
**Review process.** Abbreviations: *ICU*, Intensive Care Unit; *MET*, Medical Emergency Team; *AE*, Adverse Event.

In each hospital, the patient records will be reviewed in a multistage review process (Figure 2, based on Zegers, 2007) [5]. 

##### Stage 1: Selection of charts

A master list of eligible patients is generated at each hospital from the hospital administrative database by the quality coordinator using a uniform selection form across hospitals. Patient records selection is based on (1) an unplanned ICU admission, (2) a MET intervention or (3) a redo procedure within 24 hours for ICU patients. ICU admissions are registered on the ICUs, while MET interventions are registered on the emergency departments. Only closed patient records (after discharge from the hospital or decease of the patient) are forwarded to the next stage.

##### Stage 2: Chart review for adverse events

First, the research nurse collects from the patient records data on basic patient characteristics (gender, year of birth, reason for hospital intake, reason for transfer to ICU, number of days in the hospital prior to ICU transfer, admission day and time to ICU, number of prescribed drugs before hospital admission, ICU admission source (location/ providers of care) and outcome in ICU. The research nurse notes the data in a structured abstraction instrument, which was developed for this study.

Subsequently, each record will be reviewed by the clinical team to determine if an adverse event occurred according to the definition of Wilson (Table 2). Although each of the persons of the clinical team has a specific focus during the chart review, respectively the medical record (physician), the nurse record (research nurse) and the pharmacologic record (clinical pharmacist), assessments are made collectively. The medical records are reviewed using the structured abstraction instrument to standardize the judgements of the reviewers.

In order to evaluate the process of medical record review, data on the quality and completeness of the medical records, missing records and time measures of the screening processes are recorded. An important criterion is the recording of the actual reason for the transfer to a higher level of care.

##### Stage 3: Consensus judgment on occurrence, preventability and harm

The members of the clinical team compare their findings and come to consensus on the occurrence of an adverse event. Once the team concludes on an the occurrence of the event, the assessment on preventability and severity ratings is performed by consensus judgment.

The assessment of causation is performed using a scale from 1 to 6 (Table 3). Upon ratings of at least 4 (i.e. more than 50% likelihood), unintended injuries or complications are classified as adverse events. If the clinicians identify an adverse event, the review is continued with an assessment of its preventability using a similar six-point scale grouped into categories: no preventability, low and high evidence of preventability (Table 3). Further classification is done by type of adverse event [4,19] and patient harm (severity categories) (Table 3). The severity categories of the adverse events identified are based on the classification of the National Coordinating Council for Medication Error Reporting and Prevention (NCC MERP) [20]. The classification only includes categories E, F, G, H and I because these categories describe errors that resulted in patient harm. 

**Table 3:** Outcome measures

Determination of the presence of an adverse event is based on three criteria [4,5,10]	
1.	an unintended (physical and/or mental) **injury** which
2.	results in temporary or permanent **disability**, death or prolongation of hospital stay, and is
3.	** caused** by health care management rather than the patient's disease
To determine whether the injury is **caused by health care management** or the disease process a 6-point scale will be used [4,5,10]:	
1.	(Virtually) no evidence for management causation
2.	Slight to modest evidence of management causation
3.	Management causation not likely (less than 50/50, but 'close call')
4.	Management causation more likely (more than 50/50, but 'close call')
5.	Moderate to strong evidence of management causation
6.	(Virtually) certain evidence of management causation
The degree of preventability of the adverse events is measured on a 6-point scale, grouped into three categories [4,5,10]:	
*No Preventability*	
1.	(Virtually) no evidence for management causation
*Low Preventability*	
1.	Slight to modest evidence of management causation
2.	Management causation not likely (less than 50/50, but 'close call')
*High preventability*	
1.	Management causation more likely (more than 50/50, but 'close call')
2.	Moderate to strong evidence of management causation
3.	(Virtually) certain evidence of management causation
**Severity categories of AE’s based on the classification of the National Coordinating Council for Medication Error Reporting and Prevention (NCC MERP)**[20]**. An error occurred that:**	
–	* Category E*: contributed to or resulted in temporary harm to the patient and required intervention
–	* Category F*: contributed to or resulted in temporary harm to the patient and required initial or prolonged hospitalization
–	* Category G*: Contributed to or resulted in permanent harm
–	* Category H*: Required intervention to sustain life
–	* Category I*: Contributed to or resulted in the patient’s death (mortality rate)
**Classification of the type of AE’s**[4,19]	
–	* Operative*: an adverse event in relation to a surgical procedure or anesthesia.
–	* Procedural*: an adverse event in relation to a non-surgical procedure such as insertion of a central venous line, nasogastric tube, cardiac catheterization, etc.
–	* Anesthesia*: an adverse event in relation to anesthesia.
–	* Diagnostic*: an adverse event arising from a delayed or wrong diagnosis.
–	* Therapeutic*: an adverse event arising when a correct diagnosis was made but there was incorrect therapy or a delay in treatment.
–	* Drug/intravenous fluid*: an adverse event arising from the incorrect administration of a drug or intravenous fluid.
–	* System issue*: an adverse event in relation to problems with hospital processes such as nosocomial infection or equipment malfunction.

An expert panel of physicians is available for second advice when needed. In case of continued disagreement, an independent physician, who does not review the patient records, but only the review forms, gives the final judgment.

Case summary reports of patients that experienced an adverse event (brief narratives of the key points of each patient’s hospital stay) are written in order to facilitate an overview of the cases [21].

##### Stage 4Analysis of causes

The further analysis of the adverse events fits within a broader study that aims to explore the underlying mechanisms related to the existing safety and quality frameworks used within the hospital settings. This includes insights from the organizational-wide safety culture measurement [22]. As there is usually no single root cause, the underlying causes and contributing factors of the adverse events will be further explored using the London Protocol of Root Cause Analysis [23,24].

For each participating hospital, all the cases that were assessed by the clinical team as high preventable events are selected for further analysis. In order to conduct the analyses, additional information is collected from a variety of sources, such as for instance the availability and quality of protocols, the accessibility of information, patient identification, training of healthcare professionals, work patterns…The purpose of these analyses is to facilitate the identification of systems issues, which often relate to structure and process (both management and clinical processes) [25]. The strength of deconstructing adverse events into component elements of defaults (e.g. communication on patient information, staffing, drugs, equipment,…) lies in the fact that, once identified or characterized, potential preventive or corrective strategies can be formulated.

### Confidentiality

In this study anonymity of hospitals, health care providers and patients is of great importance. Several measures are taken to ensure confidentiality of the data.

During data collection, records are never left unattended and they are stored in a locked room or closet. Each participating hospital and each hospital admission receives a unique study number. Patient identifiers are kept in a dataset separately from the primary database. During the review process in the hospitals, the data are directly entered into a protected electronic database. The reviewers have a personal password for the electronic database. The web-based database complies with the safety and privacy requirements. Patients' names are not included in the database and after completion of the data collection and analysis, patient record identifiers are destroyed. The identity of patients or healthcare professionals will not be revealed in research reports [5].

If a reviewer has during the review process any concern about unrecognized potential deliberate harmful acts, illegal acts, or repetitive negligent behavior, these concerns will be discussed with the ethics committee of Hasselt University.

The confidentiality agreement in which the confidentiality and the rules for disseminations of results are specified, was established between the researchers, Hasselt University and the participating hospitals. Therefore, informed consent from the patients was not necessary.

### Ethical approval

Approval was obtained from the ethics committee of Hasselt University and from the ethics committee of the participant hospitals.

### Statistical analysis

The incidence of unplanned ICU (re)admissions and (preventable) adverse events requiring ICU admission will be calculated.

Primary outcomes will be measured as a rate (number of adverse events per 1000 in-hospital patient years at risk). The number of preventable adverse events (preventability rate) will be calculated as a proportion, compared with the incidence rate.

Secondary outcomes (causality, severity) are presented as incidence rates for each category.

A subgroup analysis will be performed on patient characteristics and comorbidities, type of event, location and provider of care and type of ICU.

### Testing reliability and validity

On a regular basis, the hospitals are followed up by the researchers to discuss their problems concerning the selection process of patient charts.

To test the validity of the process of screening by medical records analysts, 5% of all records are reviewed a second time by the research nurse.

### Discussion

This paper describes the rationale for a retrospective review study of adverse events that necessitate a transfer to a higher level of care. More specifically, we are particularly interested in increasing our understanding in the preventability and the root causes of these events.

There are several methodological limitations inherent to medical record review, which we are addressing within our study design.

The most important limitation is that the use of the method of medical record review itself might lead to an underestimation of adverse events. The quality of the medical records is often poor as information is missing or incomplete. Therefore, a multidisciplinary approach, in which the team is composed of a research nurse, physician, and clinical pharmacist which have experience in this area, is a key condition and adds value to conducting this chart review. A strength of our study design is the efficiency in which the members of the clinical team can focus on their own expertise. The nurse can concentrate on the nursing records, while the physician is focusing on the medical records and the clinical pharmacist is examining the medication processes. Assessments on adverse outcomes are always made collectively. In case of doubt or disagreement, a panel of physicians with different specialties is available for consultation. In addition, the clinical team assesses the completeness and usefulness of the patient charts. Incomplete records are included in the review process, as there is a higher possibility that these cases contain adverse events [15].

Second, there is the lack of an actual gold standard for adverse event detection [7]. Inevitably, the clinical team must deal with differences of medical record keeping within the participating hospitals. We therefore attempted to standardize our study protocol by conducting a pilot test in one hospital over a period of two months, in which the definitions, causality and severity ratings, abstraction instrument and the review processes were evaluated.

Third, success of this type of research is dependent on the acceptance and participation of organizations, professional groups, and individuals who may be at varying stages of readiness for investigation in this area. More specifically, the perceived threat to physician reputation or from medico-legal action should not be underestimated [19]. Therefore, the involvement of a physician might promote the acceptance of the method. Since the clinical team is composed of external researchers, almost no workload is imposed on the hospital staff and health care processes are not interrupted. Moreover, ethical approval was obtained by the ethical committees of the participating hospitals and the academic institute. An agreement was signed between the researchers, participating hospitals and the academic institute in which the privacy of the participants and the confidentiality of the data is guaranteed. It is not in the purpose of this study to compare hospitals.

Finally, although descriptive studies such as root cause analysis have limitations, they raise important challenges that will need to be overcome for future research to succeed [19]. From this perspective, we plan to obtain additional information, such as for instance the presence of protocols and accessibility to information, in order to gain additional insight in the circumstances and contributing factors leading to adverse events. Our multicenter study design allows us to aggregate data and analyze patterns of these contributing factors. Results are always interpreted within the context of the current safety management systems in the participating hospitals and recommendations will be formulated for the hospital management.

Based on this study of adverse event detection, several additional studies can be launched. It would be interesting to link the results of this study to the hospitals administrative databases to trace whether adverse events can be properly flagged. In a later time period, a cost study can be undertaken to assess the costs of care for patients with an adverse event. Insights from this study can provide information for the hospital management and policy makers to implement cost reducing interventions.

In conclusion, review of the records and further analysis of the adverse events may trigger important system changes within the hospitals.

## Abbreviations

ACHS: Australian council on healthcare standards; AE: Adverse event; ANZCA: Australian and New Zealand college of anaesthetists; APACHE: Acute physiology and chronic health evaluation; CI: Confidence interval; ICU: Intensive care unit; IHI: Institution for healthcare improvement; LOS: Length of hospital stay; MET: Medical emergency team; NCC MERP: National coordinating council for medication error reporting and prevention; PPV: Positive predictive value; UIA: Unplanned intensive care admission.

## Competing interests

The authors declare that they have no competing interests.

## Authors’ contributions

AV prepared and conceived the study protocol and the design of the study, collected and analyzed the literature and wrote the manuscript. KM conceived the study protocol and the design of the study, contributed to the manuscript and conducted the pilot study. WS contributed to manuscript, the design and conception of the study and performed the power analysis. AVL and JH contributed to the manuscript, the design and conception of the study. FW and ED has been involved in revising the article critically for important intellectual content. NC contributed to manuscript, the design and conception of the study and is the general coordinator. All authors read and approved the final manuscript.

## Authors’ information

KM is the responsible researcher for the medical record review study. AV is the responsible researcher for the Root Cause Analysis.
